# Design of the STEPS trial: a phase II randomized controlled trial evaluating eHealth-supported motor-cognitive home training for Parkinson’s disease

**DOI:** 10.1186/s12883-023-03389-y

**Published:** 2023-10-04

**Authors:** Breiffni Leavy, Jenny Sedhed, Elke Kalbe, Elisabet Åkesson, Erika Franzén, Hanna Johansson

**Affiliations:** 1 Department of Neurobiology, Care Sciences and Society, Division of Physiotherapy, Karolinska Institutet, Stockholm, Sweden; 2 Stockholm Sjukhem Foundation, Research and development unit, Stockholm, Sweden; 3 Medical Psychology | Neuropsychology and Gender Studies & Centre for Neuropsychological Diagnostics and Intervention (CeNDI), Faculty of Medicine and University Hospital Cologne, University of Cologne, Cologne, Germany; 4 Department of Neurobiology, Care Sciences and Society, Division of Neurogeriatrics, Karolinska Institutet, Stockholm, Sweden; 5 Theme Womens Health and Allied Health Professionals, Medical unit Occupational Therapy and Physical Therapy, Karolinska University Hospital, Stockholm, Sweden

**Keywords:** Parkinson’s disease, eHealth, Home exercise, Physical activity, Motor-cognitive exercise

## Abstract

**Background:**

Electronic health (eHealth) technology offers the potential to support and motivate physical activity for symptom management in Parkinson’s disease (PD). It is also recommended that motor exercise in PD be complemented with cognitive training aimed at attentional or executive functions. This paper describes the protocol for a double-blind randomized controlled trial to evaluate the effects of motor-cognitive training in the home environment, supported by eHealth.

**Methods/design:**

The Support for home Training using Ehealth in Parkinsons diseaSe (STEPS) is a double-blind single center randomized controlled trial. Two parallel groups will include in total 120 participants with mild to moderate PD who will receive either (i) the intervention (a progressive 10-week individualized motor-cognitive eHealth training with cognitive behavioral elements to increase physical activity levels) or (ii) an active control group (an individualized 10-week paper-based home exercise program). The active control group will not receive motor-cognitive exercises or cognitive behavioral approaches to increase physical activity level. The primary outcome is walking capacity assessed by the six-minute walk test (6MWT). Secondary outcomes will include gait performance during single and dual task conditions, gait speed, functional mobility and lower limb strength, balance, physical activity behavior and a range of patient reported outcome measures relevant in PD.

**Discussion:**

The STEPS trial will answer the question whether 10 weeks of eHealth supported motor-cognitive exercise in the home environment can improve walking capacity in PD when compared to a standard paper exercise program. Findings from this study will also strengthen the evidence concerning the efficacy of PD-specific eHealth interventions with a view meeting future health care demands by addressing issues of inaccessibility to specialized neurological rehabilitation in PD.

**Trial registration:**

ClinicalTrials.gov August 2022, NCT 05510739.

**Supplementary Information:**

The online version contains supplementary material available at 10.1186/s12883-023-03389-y.

## Background

 People living with Parkinson’s disease (PD) are typically less physically active than their healthy counterparts. Deteriorations in walking ability occur in line with disease progression, and reductions in gait speed and behavior are strong indicators of advancing disability [1, 2]. The merits of exercise for the treatment of gait and balance impairments in PD have been established [3–5]. Although questions remain concerning optimal exercise type, intensity and duration, high dose exercise appears to have greater effects on motor symptoms [6–8]. It is also recommended that motor training in PD be complemented with cognitive training aimed at attentional or executive functions [9]. Systematically collated evidence suggests that motor-cognitive, or dual task exercise paradigms, provide positive effects on motor features [10, 11], as well as improve dual-task performance [12]. The simultaneous combination of physical and cognitive exercise is assumed to exert synergistic responses in a process where the physical challenge ‘facilitates’ neurological responses and the cognitive challenge serve to ‘guide’ these neuroplastic processes [13, 14].

People can live with PD for several decades - a vast majority of whom live in their own home [15], and require therefore continual access to specialised neurological treatment and rehabilitation, throughout all disease stages. An added challenge for people with PD, is that sustaining exercise levels is often hindered by dopamine deficiency and evolving neuropsychiatric symptoms which in turn reduce initiative-taking [16]. Notably, despite a range of disease-related motor impairments, people with PD commonly report psychological symptoms such as anxiety, depression and low self-efficacy as major barriers to adherence to home exercise [17, 18]. It is therefore advocated that cognitive-behavioural elements be incorporated when aiming to change a person’s physical activity behaviour in the long term [19]. Such support has been successfully delivered using motivational apps in PD populations which have aimed to increase physical activity participation in daily life [20, 21].

Electronic health (eHealth) technology offers the potential to support and motivate motor-cognitive exercise as a part of everyday life with PD [22, 23]. There is also strong evidence for the efficacy of telehealth and exergame-based interventions to improve motor symptoms [24, 25]. Tablet and smartphone-based software applications have become popular means of supporting exercise among older adults and in PD [26, 27], a process further accelerated during the Covid-19 pandemic [28]. However, due to the complex nature of PD symptoms, this group requires disease-specific, as opposed to generic training applications [29]. Additionally, the core components and dose of motor-cognitive and exergame-based interventions are generally vaguely described in the literature, which inhibits interpretation and replication of findings [29]. Taking these factors into consideration, we designed a disease-specific motor-cognitive home-based intervention for PD which we subsequently tested in a feasibility study. In short, results showed the intervention to be safe and feasible in terms of recruitment capability, acceptability, and demand. Results also showed that the intensity of the level of motor challenge needed to be increased prior to efficacy testing (manuscript under review).

Here we describe the study protocol of the Support for home Training using eHealth for Parkinson’s diseaSe (STEPS) trial. STEPS is designed as a digitally supported non-supervised home-based exercise intervention. A unique element of this intervention involves the use of eHealth technology to integrate motor-cognitive exercises, which alongside cognitive behavioral techniques aims to increase physical activity behavior.

### Objectives

The primary objective of the STEPS study is to evaluate whether a motor-cognitive home exercise program, targeting functional strength and exercise capacity, can improve walking capacity in PD. Secondary objectives will be to investigate whether the intervention will result in improvements in other clinically relevant outcomes such as, physical activity participation, dual-task capacity during walking, health-related quality of life, and exercise self-efficacy. In conjunction with assessment procedures, we will also explore the added benefit of the use of inertial sensors during standard tests of gait capacity in a primary care setting.

Our underlying hypothesis is that the motor-cognitive eHealth supported home training program will be more effective than the active control condition in improving walking capacity over a 10-week period. Additionally, we hypothesize that motor-cognitive intervention will lead to improvement in dual task performance.

## Methods

### Ethical approval and trial registration

This study has been approved by the Swedish Ethical Review Authority (Dnr: 2022-02979-01 and 2023-00717-02). The STEPS trial was registered on Clinicaltrials.gov in August 2022 (NCT 05510739). All participants will receive written and verbal information regarding study details, prior to giving their written consent for participation in the study.

### Study design and setting

The STEPS trial is a randomized controlled, double blind intervention study which will occur at one clinical site. All participant assessments will occur in a primary care rehabilitation setting in Stockholm, Sweden. In Sweden, patients can receive out-patient physiotherapy treatment without doctor/specialist referral. Following assessment, participants will be randomized to either (i) the intervention (motor-cognitive eHealth training) or to (ii) an active control group (individualized home exercise program). Participants will be blinded, i.e., not made aware, of the hypothesized relative merits of the different group conditions. Training will occur in the home environment for a 10-week period. Planning and development of the STEPS study was informed by the existing literature pertaining to designing and evaluating successful mobile technologies for the management of chronic conditions among community-dwelling older adults [30]. Two specific best-practice approaches which we implemented involved application of a user-centered design, as well as use of an interdisciplinary approach. More specifically, at project onset, we recruited a group of end-users − people with PD, both men and women at varied ages and stages of the disease – who informed and gave feedback to the research group throughout the design and feasibility stages of the eHealth intervention. This iterative process informed specific decisions regarding intervention content as well as hardware and software features. Additionally, several members of the research group are physiotherapist researchers with experience of designing PD exercise interventions. We also used an interdisciplinary approach involving collaboration with Occupational therapists in a preparatory investigation of technology use among people with PD [31] and a Neuropsychologist regarding the design of PD-specific cognitive components of the motor-cognitive exercises, as well as technical experts regarding software interface aspects. The SPIRIT guidelines (2013) have guided the formation of this protocol manuscript.

### Study population

This intervention will target community-dwelling people with mild to moderate idiopathic PD, who are in need of support to increase their levels of physical activity in everyday life. This home exercise intervention is hypothesized to incur greater beneficial effects for sedentary individuals or those engaging in lower levels of physical activity [32], than that which is recommended for the maintenance of health. For this reason, recruitment advertisement materials will especially target those who are less physically active and require support to increase their physical activity levels. Inclusion and exclusion criteria (outlined in Table 1) are aimed to incorporate typical patients across a spectrum of functional capacity at mild to moderate PD stages. To ensure cognitive status enabling adherence to the study protocol, participants with severe cognitive impairment, operationalized with the MoCA, will be excluded [33]. Exclusion criteria are motivated primarily as safety measures to offset possible adverse events which can occur during unsupervised motor-cognitive training in the home-environment.

### Screening and recruitment

Participants will be recruited using a varied approach through advertisement in patient organization publications, by distributing ethically approved study flyers to neurologists/ rehabilitation practitioners and by advertisement on social media platforms managed by the responsible academic (Karolinska Institutet) and clinical sites (Primary Care Rehabilitation at Stockholm Sjukhem, Sweden). The recruitment process will occur systematically in the following sequence: Potential participants who have registered interest in the study will be contacted by study coordinators for an initial telephone interview. After the telephone screening, eligibility of potential participants will be established at an in-person clinical assessment. Prior to the assessment, potential participants will receive verbal and written information of study procedures and be given the chance to pose questions. Recruitment will occur in three to four successive waves, consisting of 30–40 participants per wave. Participants will be randomly assigned in a 1:1 ratio to the intervention or control groups. Participants in both groups will be encouraged to continue their usual daily activities during the 10-week period between measurements. They will however be advised against participating in parallel research studies aiming to affect health or adjusting their PD medication during the 10-week period.

**Table 1:** Inclusion and exclusion criteria

Inclusion criteria	Exclusion criteria
Idiopathic Parkinson’s disease	No internet connection in the home
Hoehn & Yahr stage 1 - 3	Pre-existing orthopedic or neurological diseases affecting gait
Montreal Cognitive Assessment score ≥ 21 points	Visual or hearing impairments impeding intervention delivery
Age ≥ 50 years	≥ 2 falls one month prior to inclusion
Able to walk independently indoors for six minutes without a walking aid.	

### Randomization and blinding

Randomization to the respective groups will be performed by a person in the research team who is not involved in assessment or training procedures. Allocation to the two groups will be performed in a 1:1 ratio, using web-based randomization software (randomization.com). Participants, assessors, and those performing data analysis will be blinded for group allocation. The rigor of the blinding procedure will be evaluated following post-assessment, by asking assessors to fill in a questionnaire where they report their blinding beliefs [34].

### Data collection

The testing procedure will occur at one primary care rehabilitation site and participants will be tested by physical therapists at baseline (pre-training) and at post training. Data collection will be comprised of both clinical performance tests of gait (single and dual-task), functional strength and mobility (single and dual-task), balance, cognition as well as self-reported questionnaires. All participants will be tested during the ON-phase of their medication (approx. 1–2 h following intake of anti-Parkinson medication) and testing will be scheduled to occur at the same time on both test occasions. During assessments of gait and functional mobility, participants will wear six inertial sensors (Opal, APDM Inc.) placed on feet, wrists, lumbar and sternum, to capture various spatiotemporal gait parameters.

### Primary outcome measure

The six-minute walk test (6MWT) is the primary outcome measure to assess efficacy of the intervention to improve walking capacity in PD. This test of walking capacity is reliable and valid for use in older adults and in PD [35, 36]. Participants are instructed to cover as much distance as possible, by walking back and forth between two cones placed 30 m apart, during a 6 minute interval. Participants will rate their perceived exertion and level of breathlessness using the Borg RPE and Borg CR-10 scales respectively, before and after the test [37]. When compared to healthy controls of similar age, people with PD walk at lower gait speed and with shorter stride lengths during the six-minute walk [38]. The distance covered during this test is also related to PD motor features such as bradykinesia and impaired balance [39].

### Secondary outcome measures

Gait performance during single and dual task conditions will be assessed with the 2-minute walk test (2MWT), a valid test of gait endurance in PD [40]. The single task 2MWT will be performed 10 min after completion of the 6MWT. The 2MWT will thereafter also be performed in a dual task condition during which a cognitive task, the auditory Stroop task, will be performed simultaneously while walking. During this task participants will be presented with the Swedish words for “high” and “low” in congruent (25% of stimuli) and incongruent (75% of stimuli) high and low tones via wireless headphones (Razer Thresher Ultimate). Participants will be instructed to respond verbally to the corresponding tone as quickly and correctly as possible. Verbal responses will be recorded using Audacity (version 3.2) to evaluate accuracy and reaction times. The auditory Stroop task will also be performed in a seated position in order to capture single task performance.

Gait speed will further be captured using the 10-meter Walk Test – a clinically feasible test of gait speed with proven reliability and validity in PD [41]. Participants will perform the test under two conditions, whereby they will walk a 14-meter walkway at their self-self-selected ‘usual’ gait speed and during fast walking. Testing the repeated ability to perform sit-to-stand is an easily administered clinical test which measures lower limb functional strength [42]. The five times sit-to-stand test (5TSTS) is a recommended version of this test [43], which has been reliability tested in PD cohorts and test scores also strongly relate to balance and bradykinesia in PD [44]. Functional mobility will be measured using the Timed Up and Go test [45], during both single (TUG test) and dual-task conditions (Cognitive TUG). Changes in both the amount and the intensity of physical activity behavior will be captured using the ActiGraph accelerometer model GT3X+ (Acti-Graph, Pensacola, FL, US), worn at the waist. Total daily step counts, as well as time spent (absolute and relative values) in different physical activity intensities – Sedentary behavior; Light intensity physical activity and Moderate-vigorous intensity physical activity – will be evaluated. Participants are asked to wear the accelerometer for seven consecutive days and the device will be set to sampling counts of 1-minute epochs. ActiLife software (Acti-Graph, Pensacola, FL, US) will be used to extract and process this data. Physical activity behavior will be captured at a third time interval, approximately one-year post-intervention to account for seasonal differences in weather changes as well as the achievement of long-term PA goals. Balance performance will be assessed using the 14-item Mini Balance Evaluation Systems test (Mini-BESTest) [46].

A variety of patient reported outcome measures will be assessed at both time intervals. Self-rated health and quality of life will be measured using the generic EuroQol EQ- 5D [47] and the disease-specific Parkinson’s Disease Questionnaire (PDQ-39) [48]. The EQ-5D is a non-disease-specific standardized assessment of health-related quality of life, which is widely implemented in Swedish healthcare. Respondents are required to rate their health status in relation to five dimensions; mobility, self-care, usual activities, pain/discomfort, and anxiety/depression. Self-perceived walking ability during 12 different everyday-life situations will be rated using the Swedish version of the Walking impact scale (walk 12G) [49] and participants’ balance confidence during a range of potentially challenging activities will be captured using the Activities-specific balance confidence (ABC) scale [50]. Participants’ perceived confidence, or self-efficacy to perform physical activity and exercise will be measured with the Swedish version of the exercise self-efficacy scale (S-ESS), which is reliable for use among people with neurological disease [51]. Depression and anxiety will be assessed using the Hospital Depression and Anxiety scale (HADS), which consists of two sub-scales, each incorporating 8 questions [52]. Participants will be asked to fill out all questionnaires at home. The System Usability Scale will be used to evaluate the demand and usability of the eHealth tool, [53] and perceptions of the home training programs will be investigated in both groups via a questionnaire after the 10-week period.

The Montreal Cognitive assessment (MoCA), which has good psychometric properties regarding global cognition in PD [54] will be used as a screening test for eligibility for inclusion to the study. Executive function will be assessed using Trail Making Test (TMT) II and IV from Delis Kaplan Executive Function System (D-KEFS).

### Adherence

Both intervention and control group participants will receive a home exercise diary at study outset where they note each completed training session. In addition, participants in the intervention group will also rate their level of exertion using the Borg RPE scale following each exercise session in the application. The intervention group will also note the number of steps taken each day during the 10-week period, in line with the goal of engaging in 60 min of walking per week.

### Intervention

The two training regimens in the STEPS trial will occur over a 10-week period but differ regarding intervention-targeted behavior, cognitive-behavioral elements, and dose. For an overview of respective group regimen, see Table 2.

### Intervention group condition

Participants in the intervention group will perform exercise guided by videos in a mobile application, viewed on a digital tablet three times per week. Baseline assessment will determine the level of motor difficulty (three levels) and cognitive dual task difficulty (two levels) that each participant will be assigned to. Assignment to level of motor difficulty will be decided on a case-to-case basis by the research team and be guided by participants baseline performance and (1) self-reported ability to transfer from floor to chair, (2) balance performance using the Mini-BESTest, and (3) usual gait speed measured during the 6MWT. Assignment to the level of difficulty of the cognitive dual tasks will be guided by (1) global cognition using the MoCA, and (2) performance (completion time, safety during transfers and turning, and performance on the serial subtraction task) during the TUG cog. The initially assigned levels of motor/ cognitive challenge can be altered during the training period if deemed appropriate.

Following randomization, a researcher not involved in assessments will perform a home visit or digital visit among participants to establish the safety of the home exercise environment. Short- and long-term goals regarding increasing physical activity behavior will be discussed, decided and documented during the home/digital visit. Progress towards attainment of the short-term goals will be followed up mid-intervention (5 weeks) and post-intervention (11 weeks). Commercial waist-worn pedometers (Rubicson) will be distributed for participants to track physical activity behavior (steps per day) during the intervention period. Long term physical activity goals and objective physical activity behavior will be assessed one year post intervention. The process of designing behavioral change components of the intervention was guided by an evidence-based behavioral change framework characterizing essential components of behavioral change [55] and involved elements such as, education, goal setting, goal reflection, self-monitoring and reflection, as well as digital support.

**Fig. 1:**
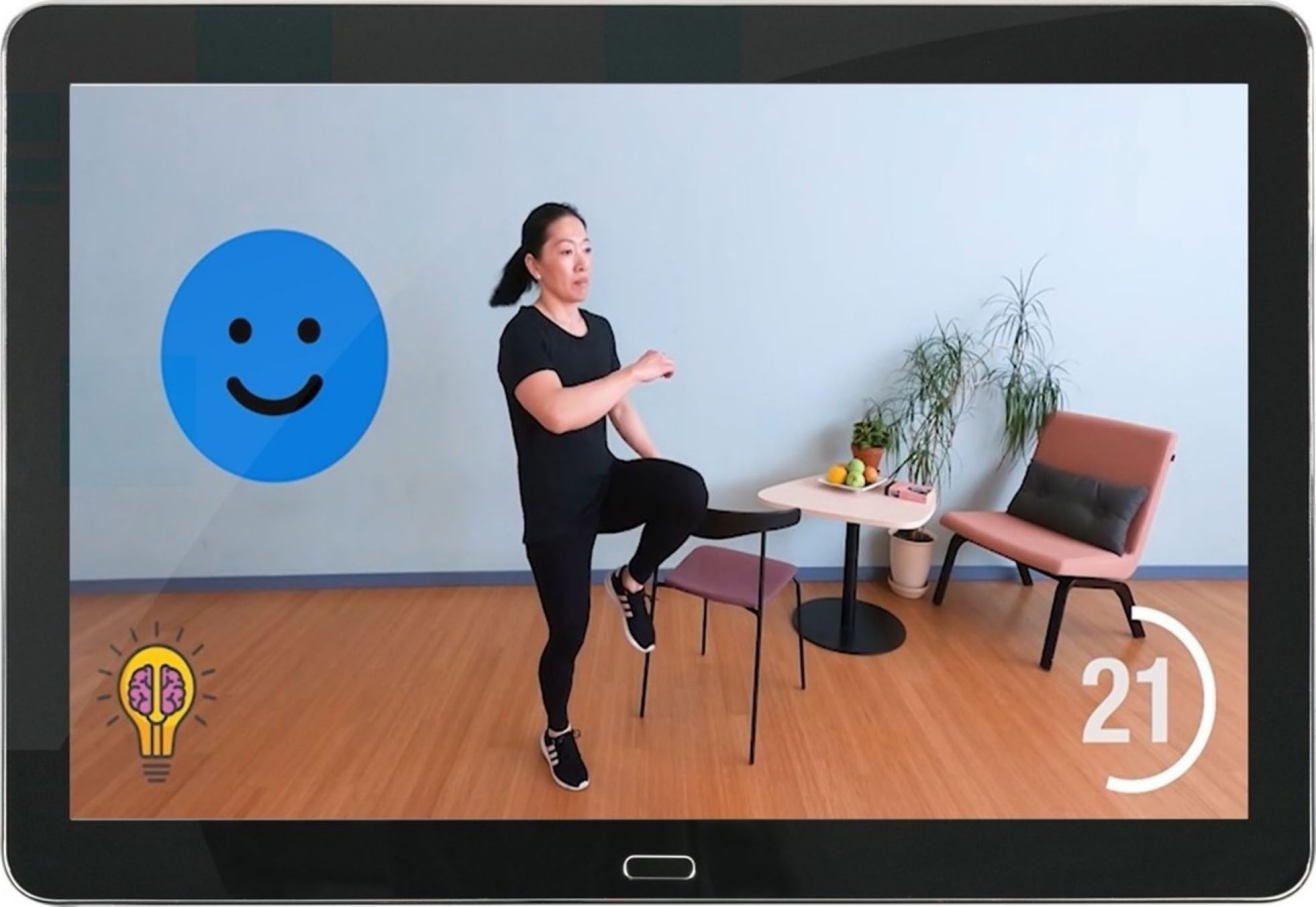
User view of a motor-cognitive exercise involving counting categories (happy blue faces) appearing on the screen (top left). The light bulb icon (bottom left) indicates the dual-task nature of the exercise, which lasts for 45 s (countdown bottom right)

All training sessions will include the components, warm-up, functional strength exercises, cardiovascular exercises, and cool-down. As of week three, the first and last session each week will also include motor-cognitive dual task elements. Figure 1 illustrates the user view of a single dual-task exercise. A schematic overview of the different types of dual-task exercises, and time spent in them can be seen in Additional file 1. The cognitive tasks were selected to specifically target the neurocognitive domains, selective attention, working memory, recognition of emotions and verbal fluency (See Additional file 2 for examples of exercise instructions, progression, and display features). During the first five weeks of the training period, weekly support will be provided using the chat function on the mobile application or via telephone, and additional support provided when needed.

**Table 2:** Description of intervention and control group regimen

	Intervention group	Control group
**Delivery mode**	Exercise Application	Paper handout
**Individual adaptation**		
* To level of motor function*	Yes	Yes
* To level of cognitive function*	Yes	No
**Exercise dose**		
* Home training sessions / week*	3	3
* Walking*	Min. 60 min / week	No
* Training period*	10 weeks	10 weeks
**Exercise progression**	Inbuilt, supported	Optional, unsupported
**Core components**		
Motor/ motor-cognitive elements:		
* Functional strength*	Yes	Yes
* Flexibility*	Yes	Yes
* Cardiovascular*	Yes	No
* Cognitive dual tasks*	Yes	No
Cognitive-behavioral components:		
* Short & long-term PA goal setting*	Yes	No
* Self-monitoring and reflection*	Yes	No
* Educational/motivational videos*	Yes	No
* Remote support* via video / phone	Yes	No

### Control group condition

Participants in the control group will receive a paper printout with home exercises to be performed three times per week. The home exercise program will be instructed by a physiotherapist at an in-person visit scheduled after baseline assessments. Level of motor difficulty (three levels) will be decided by the same criteria as in the intervention group. The exercises will focus on functional strength, flexibility, and balance. Written information on how exercises can be progressed will be included, but no support for progression will be given during the training period. Should participants in the active control group have further questions or concerns regarding their exercise program, they will be able to contact their physical therapist by telephone.

### Power and sample size estimate

A sample size calculation was conducted to determine the number of participants needed to evaluate an ‘intervention group’ compared to an ‘active control group’. A previous study investigating functional limitations and task performance among people with mild and moderate Parkinson disease has reported 6MWT performance of 460.24 ± 107.09 and 397.81 ± 104.02 (mean ± standard deviation) [56].

Assuming a mean and standard deviation of 460.24 and 107.09 m respectively, the study would require a sample size of: 58 participants for each group (a total sample size of 116, assuming equal group sizes), to achieve a power of 80% and a level of significance of 5% (one sided), for detecting an intervention effect (difference between treatments) in means between the intervention and the control group of 50 m. Considering a 5% drop-out, the study would require a sample size of: 60 participants in each group (a total sample size of 120). The calculations were done using a two sample mean test, one sided test, in STATA version 17.

### Statistical analysis plan

Analysis will be performed based on intention to treat and per protocol basis. The two-sided 0.05 level will be applied for significance in all analyses, except the main outcome 6-minute walk test, which is tested with a one-sided test.

Baseline demographics and other patient’s characteristics will be tabulated and summarized by treatment group. Continuous variables will be described by standard descriptive statistics (mean, standard deviation, median, interquartile range, minimum, and maximum), and categorical variables will be summarized by frequency tables with number and proportion in each category (with the corresponding sample sizes).

In the primary analysis of the effect of STEPS intervention to improve walking capacity in comparison to the active control condition a linear mixed model (LMM) with subject-specific random intercept will be specified to assess possibles changes in 6MWT performance over time within and among groups. LMM accounts for both fixed and random effects, can analyze data with repeated measures as well as can handle missing values. Specifically, the LMM will include treatment, time, levodopa equivalent dose and time*treatment interaction term as fixed effects, and subject/participant as a random effect. An unstructured correlation matrix will be used. Missing values will be imputed as part of the LMM model assumptions. A linear regression model will be adjusted for studying the influence of baseline demographics, patient’s medical history variables and treatments on the change in 6MWT performance.

In terms of the secondary outcomes, for continuous variables, linear mixed models (LMM) with subject-specific random intercept will be specified to assess possibles changes over time within and among the intervention and control groups. The strength of the effect size will be calculated using Cohen’s d for all outcomes that are normally distributed. For dichotomous variables, change in proportions within groups at end of treatment with respect to baseline (pre-treatment vs. post-treatment) will be assessed using McNemar’s test. For categorical variables, frequency distributions will be estimated and compared between the groups using Chi-squared test or the exact Fisher test.

### Adverse events

We estimate that the risks of participating in this study are small and are equivalent to those involved when engaging in everyday physical activities in the home environment and in everyday life. Increased engagement in physical activity among older adults with gait and balance impairments implicitly involves an increased risk exposure for falls and related injuries. Both the control and intervention group exercise programs will be individually adapted in relation to participants baseline function regarding functional and dual-task capacity, thereby minimizing eventual risk for falls in both group conditions. Additionally, no adverse events occurred during the feasibility testing of this intervention among a similar group of target participants (manuscript under review).

Adverse events will be systematically monitored by the research group throughout the 10-week intervention period. Participants will be asked to document in their home exercise diary any falls, near-falls or harmful events which occur. Participants in both the intervention and control groups will also receive a contact number to study staff in the occurrence of a fall or injury. All details of adverse events which occur will be documented according to patient safety procedures at the rehabilitation clinic at the Stockholm Sjukhem foundation.

## Discussion

Our objective with the STEPS trial is to determine if a home-based eHealth intervention with an added cognitive-behavioral approach is more effective than an active control intervention in improving walking ability among people with PD. The intervention is unique in its design and the development of which has been guided by best practice recommendations [57]. If efficacy of this intervention is proven this will strengthen the evidence base concerning eHealth interventions which enable the self-management of PD symptoms in everyday life. Access to evidence-based digital rehabilitation is crucial to meet the future demands on health care considering demographic changes and a limited availability of health care resources. Additionally, the parallel evaluation of a broad range of patient-centered outcomes will inform the future selection of clinically meaningful digital-outcome measures among this patient group [58].

### Accessibility

This unsupervised eHealth intervention also aims to address issues of inaccessibility to specialized neurological rehabilitation for people with PD. Cohort studies in western countries report a lack of accessibility to specialist neurologist care [59], and that a minority (14–40%) of people with PD receive physiotherapy treatment [15, 60]. This inaccessibility to specialized care is presumed higher in rural areas and in non-Caucasian cohorts. Use of technology to deliver rehabilitation in the home offers accessibility and convenience that may help bridge disability-related barriers to clinic-based treated which factor in PD [61].

Findings from this trial will strengthen the knowledge base and guide the current shift in health care models from clinic-based to a more accessible telerehabilitation [62]. Furthermore, by delivering the STEPS intervention in a primary care context this study addresses the challenge of integrating eHealth rehabilitation into current health care systems [61]. Intervention design also addresses known cognitive barriers for engaging in physical activity in PD such as lack of exercise, self-efficacy, and motivational ability by integrating cognitive behavioral approaches. Motivational strategies delivered in the home should promote exercise becoming an embedded feature of everyday activities [63], and thereby support long-term disease management.

### Supplementary Information

**Additional file 1.**

**Additional file 2.**

**Additional file 3.**

## Data Availability

Due to Swedish and EU personal data legislation datasets generated during current study will not be made publicly available, but availability may be permitted by the corresponding author on reasonable request. Any sharing of data will be regulated via a data transfer and user agreement with the recipient.
