# Supplementary figures and images for: Design of the STEPS trial: a phase II randomized controlled trial evaluating eHealth-supported motor-cognitive home training for Parkinson’s disease
Source: BMC Neurol. 2023 Oct 4;23:356. doi: 10.1186/s12883-023-03389-y (PMC10548709; doi:10.1186/s12883-023-03389-y)

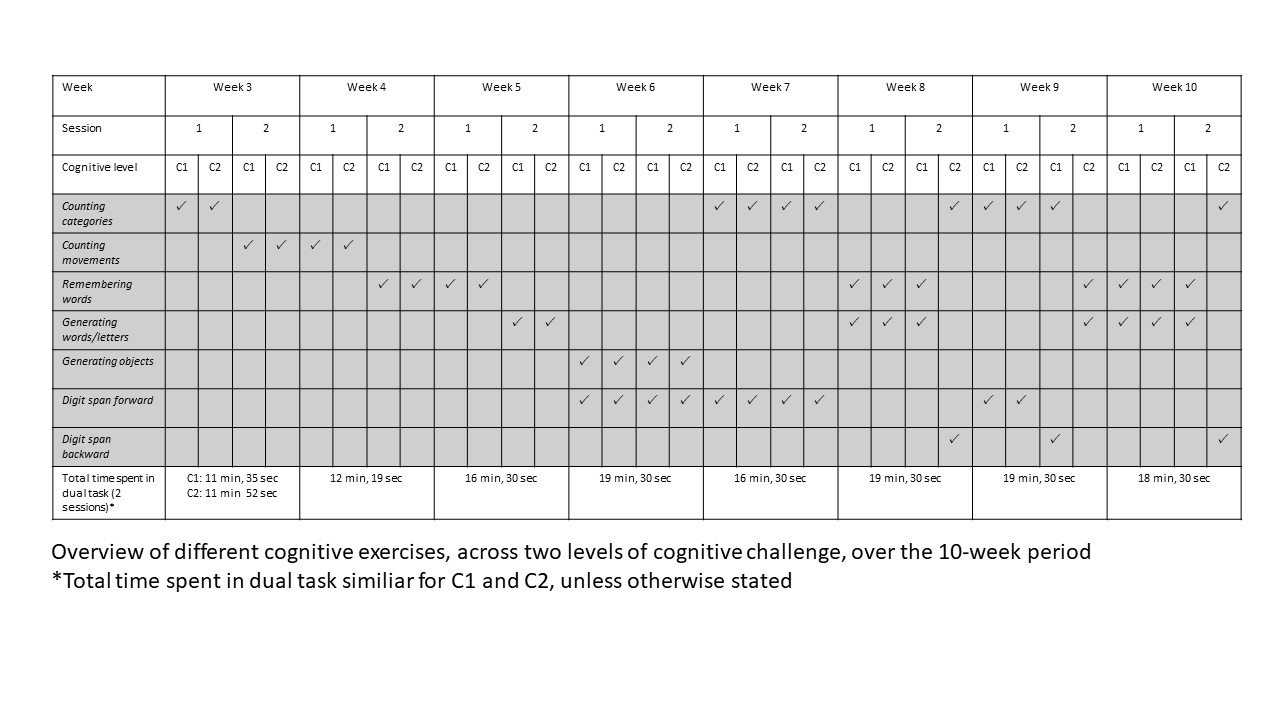

Supplement: Supplementary file 1 — Additional file 1. [file 12883_2023_3389_MOESM1_ESM.jpg]

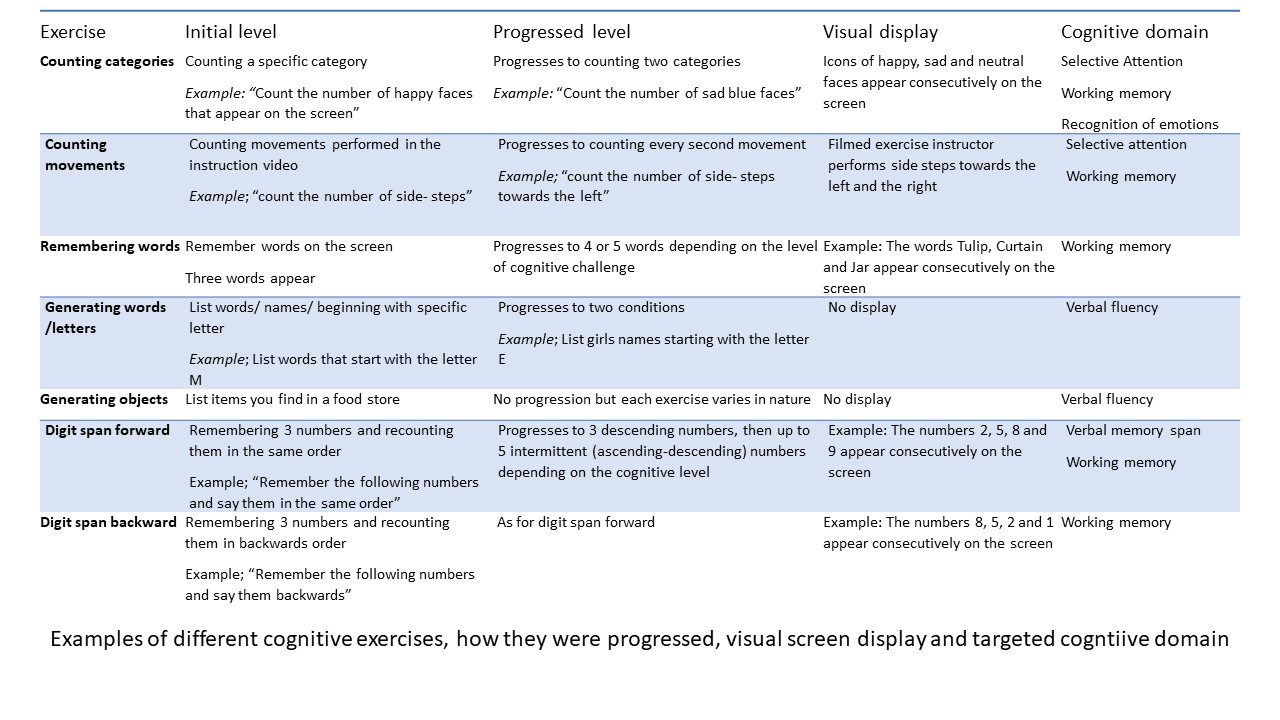

Supplement: Supplementary file 2 — Additional file 2. [file 12883_2023_3389_MOESM2_ESM.jpg]
